# The General Public Knowledge, Attitude, and Practices Regarding COVID-19 During the Lockdown in Asian Developing Countries

**DOI:** 10.1177/0272684X211004945

**Published:** 2021-04-08

**Authors:** Sikandar Ali Qalati, Dragana Ostic, Mingyue Fan, Sarfaraz Ahmed Dakhan, Esthela Galvan Vela, Zuhaib Zufar, Jan Muhammmad Sohu, Jinlan Mei, Troung Thi Hong Thuy

**Affiliations:** 1 Department of Management, School of Management, Jiangsu University, Zhenjiang, P.R. China; 2 Department of International Trade, School of Finance and Economics, Jiangsu University, Zhenjiang, P.R. China; 3 Department of Information Management and Information System, School of Management, Jiangsu University, Zhenjiang, P.R. China; 4 Department of Business Administration, Sukkur IBA University, Sindh, Pakistan; 5 Escuela de Administracion y Negocios, CETYS Universidad, Tijuana, México; 6 School of Law, Jiangsu University, Zhenjiang, P.R. China; 7 School of Education, Minnan Normal University, Zhangzhou, P.R. China

**Keywords:** knowledge, attitude, practices, developing countries, the general public, lockdown, COVID-19

## Abstract

The recent outbreak of coronavirus disease (COVID-19) is the worst global crisis. Since no successful treatment and vaccine have been reported, efforts to improve the public's knowledge, attitudes, and practices are critical to reducing the spread of COVID-19. This study aims to investigate the general public knowledge, attitude, and practices regarding COVID-19. A cross-sectional online survey was conducted in three developing countries (China, India, and Pakistan). The reason for choosing only three countries is to identify the cross-border effect statistically and data collection constraints. The IBM SPSS version 23.0 was used for descriptive, univariate, and multivariate analysis of the study. One thousand one hundred and sixty participants completed the study, one-quarter of them were female, and three-quarters were male. The study's findings evidenced that the knowledge and attitude correlation was 58.4% and between knowledge and practices 18.2%. Furthermore, the knowledge was found lower in females, among India and Pakistan, and people aged less and equivalent to 30 years. The attitudes among respondents were found poorer among unmarried females and India and Pakistan residents. While the practices found lower among employed, unemployed and, respondents had a bachelor's degree, and females reside in India. And future studies should focus on factors that influence the government regarding the imposition of lockdown, boost the economy in the pandemic, and motivate the general public to follow the health institution's instructions.

COVID-19 started from Wuhan city China and has currently afflicted almost the entire world.^1^ Coronavirus was also identified back in the years of 2003 and 2015 named as Severe Acute Respiratory Syndrome-Coronavirus (SARS-Cov) and Middle East Respiratory Syndrome-Coronavirus (MERS-Cov), which were similar to COVID-19 and these exhibited similarities to COVID-19 was first reported at the end of December 2019.^2^ At present, 1st November 2020, COVID-19 confirmed cases reached 45,678,440 and deaths over 1,189,945. Europe and the Americas have reported most infected regions of the world with over 20,477,535 and 10,803,232 confirmed cases, respectively, whereas Western Pacific and Africa 733,828 and 1,319,279 had less confirmed cases, respectively.^3^ The developed countries, especially the United States of America (91,99,205), Brazil (55,35,605), Russian Federation (16,06,267), and France (13,73,036), have had more confirmed cases as compare to developing countries China (84,450), India (81,37,119), and Pakistan (3,33,970). Developed countries had enough resources to face this pandemic because of the strong economy and resources. In contrast, developing countries were not ready due to their unstable economy and lack of basic resources to the public. It was a big challenge for developing countries to respond to the situation. On 14th April 2020, the world health organization published the COVID-19 strategy report focused on what we have learned from a pandemic. One of the important lessons and recommended strategies to fasten the tracking of suspected cases of COVID-19 is isolation and the imposition of lockdown to restrict the spread of COVID-19.^4^

COVID-19 is transmitted through inhalation of aerosols from an infected person.^5^ Mainly patients with pre-existing illnesses (such as hypertension, diabetes, lung disease, cancer, and cardiac disease), kids, and old age have been identified as potential risk determinants for severe disease and morality.^6^ To date, there is no antiviral vaccine or treatment that has been proposed for COVID-19.^7^ Further information related to its transmission, distribution, prevention, treatment, and pathophysiology is being studied. The world health organization proposed preventing person-to-person transmission by preventing close contacts and healthcare workers from being infected.^8^ On 13th October 2020, WHO stated that the primary protection measures to reduce the risk of COVID-19 infection and its spread includes social distancing, regular handwashing, using sanitizer, and respiratory hygiene (covering nose and mouth while sneezing or coughing).^8^ Furthers, the general public needs to maintain at least a 1-meter distance to reduce the risk of infection and disease spread. Besides, make wearing a mask a normal part of being around the people. Further, avoiding 3Cs (spaces that are closed, crowded, or involving close contact), meet people outside rather than indoor ones.^8^

Globally, Along with WHO, most of the government favoured and imposed drastic lockdown at an early stage of COVID-19 because it is considered one of the best and recommended approaches to control the current pandemic; it could be more effective if executed with integrity. The governments of Pakistan and India initially announced two weeks and then three weeks lockdown, respectively, which was decided to extend due to the increased number of cases. The lockdown decision was not easy for the governments because it was supposed to affect the economy and the public badly. Along with major issues, the general public also faced sleep disturbance problems, anxiety, and depression due to lockdown.^9^ The general public in India faced job loss, and a decline in income; the health care was increased among the nation, and there was a decrease in food supply.^10^ And the extension of lockdown created further challenges to an already distressed population (largely daily-wages workforce) and to ensure strict compliance with social distancing guidelines.^11^ Mohamad and colleagues' study concluded that lockdown measures' effectiveness is largely based on the compliance and cooperation of all members of society.^12^ Furthers, to decrease the adverse effects of COVID-19, there is a need to increase the general public's knowledge so that spread of the disease can be reduced. Therefore, the knowledge, attitude, and practices people possess regarding COVID-19 play a key role in determining a society’s readiness to accept behavioral change measures from the health authorities. Similarly, several studies dedicated to understanding the knowledge, attitude, and practices regarding COVID-19 in different countries.^13–16^ Measurement of the public's knowledge, attitudes, and practices will help provide a better understanding of the COVID-19 and the establishment of health-promoting advertisement and preventive strategies. Besides, poor understanding of COVID-19 in the general public can result in delayed identification and can be a key factor in the rapid spread of disease.

The lessons learned from the previous outbreak are that knowledge and attitudes are linked with the level of emotions and panic, which may further complicate the parameters to contain the spread of COVID-19. This study survey provides a general picture of three Asian developing countries COVID-19 prevention practices, which may help governments address future health crises comprising infectious diseases.

## Literature Review

Globally, the COVID-19 pandemic has become a major concern despite developed or developing countries; it is one of the most dominant challenges globally. Countries worldwide adopted and still adopting unprecedented infection prevention and control measures to urgently curtail the transmission of COVID-19. As aforementioned, COVID-19 was first reported by WHO on the 31st December 2019 and announced a global pandemic on the 11th March 2020.^17^

Several studies have been conducted associated with COVID-19 effects on global economies, individuals, and communities.^18^ studied a sample of 30 countries, and estimated a decline of over 10 to 15% in GDP. Further, it is stated that service-oriented economies will be negatively affected and may have more job risks. And countries highly dependent on foreign trade are even more negatively affected. Similarly, Nicola et al.^19^ predicted that social distancing, travel restriction, and social isolation might lead to a reduced workforce in most sectors and caused unemployment.

The COVID-19 outbreak is severely disrupting the world economy. Almost all the economies are struggling to slow down the disease's spread by imposing lockdowns, quarantining suspected cases, testing and treating patients, etc. COVID-19 has a great effect on society and the global environment.^20^

Despite the effects on society, the global environment, and the economy, some studies have investigated the knowledge, attitude, and practices/behavior of individuals regarding COVID-19. For instance, Honarvar et al.^14^ investigated individuals' knowledge, attitude, risk perception, and practices of an adult towards COVID-19 in Iran. Similarly, Reuben et al.^16^ also measures the knowledge, attitudes, and practices towards COVID-19 among people in North-Central Nigeria.^13^ also evidenced that individuals having sound knowledge exhibits a positive attitude and adopted practices to lessen the spread of COVID-19.

## Methods

### Study Design, Population, and Data Collection

An online cross-sectional survey was used from 16th to 31st October 2020 among the three Asian countries in the English and Chinese languages. An online survey was created due to the state of the pandemic,^21,22^ using Google form, and the link was shared using social media applications (i.e., WeChat, WhatsApp, Facebook, and emails) to reach a large number of valid participants in the general public.^23^ A sample size of the study estimated by using formula (*n*=*Z*^2^/4*d*^2^), where *n* = sample size, *Z*=level of confidence interval 95.0%, *z* = 1.96, *d*= tolerated margin of error, we used 3% margin of the error, hence *n*= (1.96)^2^/4(0.03)^2^ = 1067.^24,25^ Therefore, the minimum sample size to conduct this study is 1067. However, a larger sample size of 1161 was collected. Given the ethical concern, respondents were assured that their participation would remain voluntary, confidential, and anonymous.

### Survey Questionnaire

The closed-ended questionnaire consisted of four sections: the first section include 6 demographic items and one general question regarding activities during the lockdown, and the second, third, and fourth sections consisted of 24 questions: 6 knowledge-based, 9 attitudes-based, and 9 practice-based, respectively. There are several conceptual models in health behavior research, for instance, the behavior change wheel and the health belief model.^26–28^ We selected the health belief model as the main conceptual framework, designed a questionnaire based on the health belief model, and followed prior studies advocating using the health belief model to ask questions for knowledge, attitude, and practice regarding COVID-19.^14^ Demographic items include country, gender, age, education, employment, and marital status.

The second section includes the six items to assess the knowledge of the public related to COVID-19. Dichotomized “yes” and “no” options were used to assess six items. The total score of knowledge was up to 6, and in SPSS coded as (0 = No, 1 = Yes). The third section assessed the attitudes of the general public about COVID-19. For scoring of attitude, 9 items were scored on a 5-point Likert scale (1 = strongly disagree, 5 = strongly agree) with a maximum score of 45. The fourth section includes 9 items that evaluate the public's practices towards COVID-19; questions were scored on a 5-point Likert scale (1 = never, 5 = always) with a maximum score of 45. Every appropriate answer added five marks to a participant's total score and one given for inappropriate. We considered if the achieved score was less than 40%, 40–70%, and over 70% of total achievable scores in knowledge, attitude, and practice section as inappropriate, roughly appropriate, and appropriate, respectively following Honarvar et al.^14^ This study adopted scales from prior studies.^14,23,29^ Table 1 reveals the reliability of the scale used. Scholars used Cronbach's Alpha to check the reliability of the scale. According to Nunnally,^30^ Cronbach’s alpha values should exceed 0.7: the threshold values of constructs in this study ranged between 0.729 to 0.928.

**Table 1.** Reliability Statistics and Test of Normality.

	Cronbach’s alpha	Items	Kolmogorov-Smirnov			Shapiro-Wilk		
Overall	0.890	24	Statistic	*df*	Sig.	Statistic	*df*	Sig.
Knowledge	0.729	6	0.332	1160	0.19	0.734	1160	0.72
Attitude	0.801	9	0.107	1160	0.12	0.908	1160	0.81
Practice	0.928	9	0.194	1160	0.14	0.839	1160	0.79

Quality assurance was accomplished by supervision on the data accumulation and data extraction process, data entry to software, and data analysis. Furthermore, this study also evidenced the normality of data (Table 1). Data normality was analyzed,^31^ using normality test^32,33^ and found that the present study meets a normal distribution as significance level is greater than 0.05 (Table 1). Normality test as Kolmogorov-Smirnov and Shapiro-Wilk also specified significant Gaussian distribution. Thus, Independent samples T-test and ANOVA tests were employed to execute data.

### Statistical Analysis

IBM SPSS version 23.0 was used for statistical data analysis. Univariate construct analysis was performed using *t*-test and analysis of variance (ANOVA). The Pearson coefficient test evaluated the correlation between knowledge, attitude, and practices. Also, multivariable analysis was performed using linear regression after taking into account the VIF and tolerance levels. In this study, *t*-value >1.96, and *p*-value <0.05, was considered to be a significant level.

## Results

One thousand one hundred and sixty persons from three Asian countries completed the survey. Table 2 demonstrates the full overview of the demographic characteristics of the participated individuals. Out of 1160 respondents, 712 (61.3%) were aged < 30 years, and nearly 38.7% (448) were aged over 30 years, the male-to-female ratio was 2.53, and 66.9% were single. The majority of respondents had a master's degree, 624 (53.8%), .and 46.2% were employed. A vast of participants were from India (n = 424, 36.5%).

**Table 2.** Demographic Characteristic of Participants.

Characteristics	
Age (year), *n* (%)	
≤30	712 (0.61)
>30	448 (0.39)
Gender, *n* (%)	
Male	832 (71.7)
Female	328 (28.3)
Marital status, *n* (%)	
Single	776 (66.9)
Married	384 (33.1)
Education, *n* (%)	
High school	128 (11)
Bachelor	280 (24.2)
Master	624 (53.8)
PhD	128 (11)
Employment status, *n* (%)	
Student	464 (40)
Employed	536 (46.2)
Unemployed	88 (7.6)
Retired	72 (6.2)
Country, *n* (%)	
China	388 (33.5)
India	424 (36.5)
Pakistan	348 (30)

**Figure 1.**
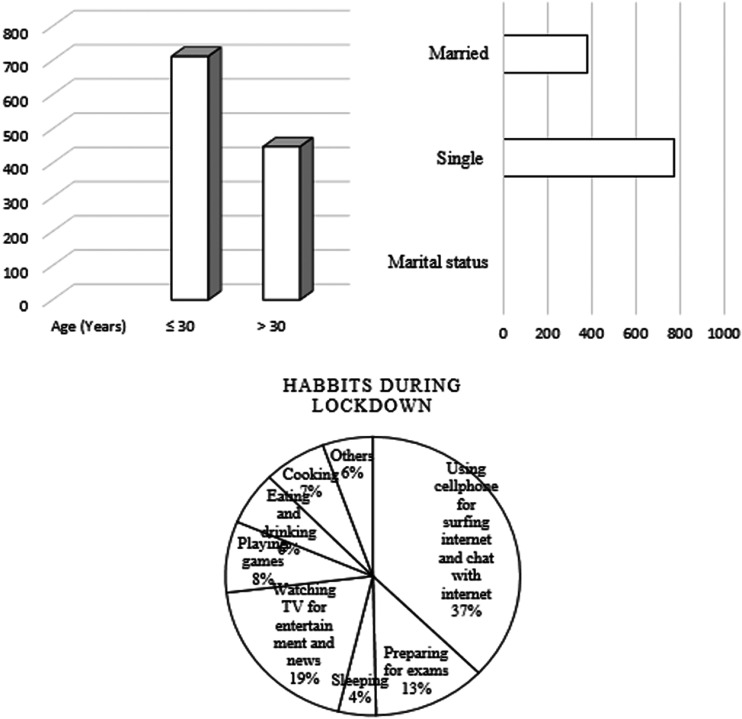
Graphical Representation of Age, Marital Status, and Habits During Lockdown.

Figure 1 cumulatively shows the graphical representation of age, marital status, and participants' habits during a lockdown. Most of the respondents, 37.0% (432), reported the use of cell phones to surf the internet or to chat with friends. And 224 (19.0%) of them watch TV for entertainment and news purpose.

The most frequent correct response to the knowledge questions was about quarantine (93.8%), avoid close contact with infected person 1080 (93.1), avoid going to crowded places (87.6%), and the last one was about the isolation of COVID-19 patient (67.4%) to ensure effective implementation of infection control measures (Table 3).

**Table 3.** Knowledge-Based Participant’s Response Among Three Asian Countries.

No	Item	No (inappropriate)	Yes (appropriate)
K1	Do you know a minimum incubation period of COVID-19 is 2–14 days?	72 (6.2)	1088 (93.8)
K2	Do you know COVID-19 mainly spread through close contact with an infected person?	80 (6.9)	1080 (93.1)
K3	Do you know runny rose, cough, and fever are symptoms of COVID-19?	152 (13.1)	1108 (86.9)
K4	Do you know COVID-19 spreads via respiratory droplets of infected individuals?	296 (25.5)	864 (74.5)
K5	Do you know to prevent COVID-19, individuals should avoid going to crowded places such as the bus and railway station, shopping, and four public places?	144 (12.4)	1016 (87.6)
K6	Do you know the isolation of COVID-19 patients is critical to ensure effective implementation of infection control measures?	376 (32.4)	784 (67.4)

The knowledge and attitude had a correlation of 0.584 (*p*-value < 0.001). In response to attitude (720 + 232), 82.0% believe that washing hands with soaps and sanitizer can prevent transmission of COVID-19, 81.37% believe evidenced that reporting symptoms of runny nose, cough, and fever to local health authorities is essential to prevent further transmission of COVID-19. Less than half of respondents (18.6 + 26.9), 45.51%, observed less preventive measures against COVID-19 in the community, and (56 + 11 + 296) 41.3% did not agree that lockdown saves lives (Table 4).

**Table 4.** Attitude-Based Participant’s Response Among Three Asian Countries.

No	Item	Strongly disagree, *n* (%)	Disagree, *n* (%)	Neutral, *n* (%)	Agree, *n* (%)	Strongly agree, *n* (%)
A1	To what extent do you agree with the government lockdown decision?	32 (2.8)	136 (11.7)	256 (22.1)	600 (51.7)	136 (11.7)
A2	To what extent do you agree with the preventive measures against COVID-19 observed in the community?	136 (11.7)	288 (24.8)	208 (17.9)	216 (18.6)	312 (26.9)
A3	To what extent do you agree that lockdown save lives?	56 (4.8)	128 (11)	296 (25.5)	448 (38.6)	232 (20)
A4	To what extent do you think that media plays an important role in spreading accurate information related to COVID-19?	32 (2.8)	128 (11)	216 (18.6)	552 (47.6)	232 (20)
A5	To what extent do you agree that there is a risk of catching COVID-19 infection during a lockdown?	56 (4.8)	112 (9.7)	216 (18.6)	552 (47.6)	224 (19.3)
A6	To what extent do you agree that washing hands with soaps and sanitizers can prevent transmission of COVID-19?	16 (1.4)	64 (5.5)	128 (11)	720 (62.1)	232 (20)
A7	To what extent do you agree that avoiding contact with ill people having symptoms of runny nose, cough, and fever can prevent the spread of COVID-19?	8 (0.7)	40 (3.4)	200 (17.2)	528 (45.5)	384 (33.1)
A8	To what extent do you agree that COVID-19 is a dangerous and deadly disease?	16 (1.4)	72 (6.2)	192 (16.6)	544 (46.9)	336 (29)
A9	To what extent do you agree that reporting symptoms of runny 0se, cough, and fever to local health authorities is essential to prevent further transmission of COVID-19?	40 (3.4)	48 (4.1)	128 (11)	672 (57.9)	272 (23.4)

The knowledge and practices had a correlation of 0.182 (*p*-value < 0.001). In terms of practices, the most frequent appropriate practice was the teaching COVID-19 prevention 74.4%, and the least frequent one was disinfection of hands and sanitizing 54.4%. Furthermore, 73.7% reported that they follow news related to COVID-19, 73.1% practiced keeping 1–1.5 m distance, 72.4% had increased avoiding contact with others (refer to Table 5).

**Table 5.** Practice-Based Participant’s Response Among Three Asian Countries.

No	Item	Never, *n* (%)	Rarely, *n* (%)	Some times, *n* (%)	Often, *n* (%)	Always, *n* (%)
P1	How often do you cover your mouth when coughing and sneezing?	80 (6.9)	128 (11)	144 (12.4)	560 (48.3)	248 (21.4)
P2	How often do you follow the news of COVID-19?	80 (6.9)	96 (8.3)	128 (11)	632 (54.5)	224 (19.3)
P3	How often do you wear a surgical mask while going outside?	128 (11)	96 (8.3)	176 (15.2)	528 (45.5)	232 (20)
P4	How often do you avoid contact with others while having symptoms of runny nose, cough, and fever?	104 (9)	96 (8.3)	120 (10.3)	648 (55.9)	192 (16.6)
P5	How often do you avoid direct contact with the mouth, nose, and eyes?	104 (9)	112 (9.7)	128 (11)	560 (48.3)	256 (22.1)
P6	How often do you hug and shake hands with others?	48 (4.1)	144 (12.4)	136 (11.7)	632 (54.5)	200 (17.2)
P7	How often do you keep at least 1–1.5 distance from others?	40 (3.4)	128 (11)	144 (12.4)	576 (49.7)	272 (23.4)
P8	How often do you try to teach other people about the prevention of COVID-19?	48 (4.1)	104 (9)	144 (12.4)	624 (53.8)	240 (20.7)
P9	How often do you wash hands or disinfect your hands?	144 (12.4)	176 (15.2)	208 (17.9)	400 (34.5)	232 (20)

Table 6 reveals the univariate analysis of the study. It shows that females had a lower level of appropriate knowledge, attitude and are practicing more inappropriate practice comparative to males. Individuals with age less than or equivalent to 30 years also had a lower level of knowledge and attitude than the elder. Still, the comparison is insignificant, and practices are also inappropriate among them comparative to elders. The educated people had an appropriate level of knowledge, attitude, and practices compared to people with high school and bachelor levels of education. Still, the comparison of practices among the group is insignificant. The married participants had an appropriate level of knowledge, attitude, and practices than singles. The students had a lower level of knowledge compared to other occupants, in attitude retired had inappropriate one, while in practice employed and unemployed had poorer practices. Among the countries, China had a sound or higher level of knowledge, attitude, and practice than India and Pakistan.

**Table 6.** Univariate Analysis of Knowledge, Attitude, and Practices Based on Demographics.

Variable	Knowledge (6)			Attitude (45)			Practices (45)		
	Mean ± SD	Statistics	*p*-Value	Mean ± SD	Statistics	*p*-Value	Mean ± SD	Statistics	*p*-Value
Gender									
Male	5.19 ± 1.25	*t* = 5.80	<0.001	34.00 ± 4.945	*t* = 2.827	<0.005	33.43 ± 7.48	*t* = 3.96	<0.001
Female	4.63 ± 1.56			32.12 ± 6.722			31.17 ± 9.18		
Age (year)									
≤30	5 ± 1.42	*t* = –1.47	0.142	33.47 ± 5.22	*t* = –1.590	0.112	31.99 ± 8.78	*t* = –5.13	<0.001
>30	5.10 ± 1.28			34.01 ± 5.97			34.21 ± 8.78		
Education									
High school	4.50 ± 1.70	*F* = 34.10	<0.001	33.37 ± 4.72	*F* = 3.512	0.015	33.50 ± 5.87	*F* = 1.756	0.154
Bachelor	4.54 ± 1.64			32.15 ± 6.48			31.91 ± 9.58		
Master	5.24 ± 1.09			33.48 ± 5.26			32.91 ± 7.67		
PhD	5.62 ± 0.99			34.62 ± 5.093			33.43 ± 8.08		
Marital status									
Single	4.91 ± 1.44	*t* = –4.50	<0.001	33.60 ± 5.65	*t* = –0.66	0.505	32.31 ± 8.46	*t* = –3.02	<0.001
Married	5.27 ± 1.15			33.83 ± 5.27			33.75 ± 7.10		
Employment status									
Student	4.87 ± 1.65	*F* = 3.50	<0.015	33.39 ± 6.35	*F* = 0.798	0.495	33.74 ± 8.06	*F* = 6.97	<0.001
Employed	5.11 ± 1.17			33.82 ± 5.22			31.70 ± 8.66		
Unemployed	5.18 ± 0.94			33.47 ± 3.31			32.81 ± 6.24		
Retired	5.22 ± 0.922			32.95 ± 3.98			34.8 ± 2.31		
Country									
China	5.56 ± 1.1	*F* = 61.69	<0.001	36.23 ± 3.929	*F* = 100.5	<0.001	34.08 ± 8.38	*F* = 15.17	<0.001
India	4.50 ± 1.70			30.91 ± 6.68			30.95 ± 8.81		
Pakistan	5.03 ± 1.06			33.44 ± 4.38			33.30 ± 6.72		

According to multivariate analysis, knowledge was found lower in females, among India and Pakistan, and people aged less and equivalent to 30 years (Table 7). The respondents' attitudes were found poorer among unmarried people, females, and the residents of India and Pakistan (Table 7). While the practices found poorer among employed, unemployed and, respondents had a bachelor's degree, and females reside in India (Table 7).

**Table 7.** Linear Regression Analysis of Participants Characteristics of Knowledge, Attitude, and Practices.

Knowledge						Attitude					Practice				
Variables	Unst: B	St: B	*t*-value	*p*-value	CI for B	Unst: B	St: B	*t*-value	*p*-value	CI for B	Unst: B	St: B	*t*-value	*p*-value	CI for B
(Constant)	4.32		24.93	<.001	3.98, 4.66	34.49		47.01	<.001	33.0, 35.93	33.62		31.31	<.001	31.51, 35.72
Gender	–0.54	–0.17	–6.36	<.001	–0.70, –0.37	–1.14	–0.09	–3.18	<.001	–1.85, –0.44	–1.85	–0.10	–3.52	<.001	–2.88, –0.82
Country	–0.2	–0.13	–4.54	<.001	–0.30, –0.12	–1.13	–0.17	–5.69	<.001	–1.52, 0.74	–0.08	–0.09	–0.30	0.761	–0.66, 0.48
Age	–0.13	–0.04	–1.63	.102	–0.28, 0.02	0.20	0.018	0.612	0.541	–0.45, 0.87	2.12	0.12	4.29	<.001	1.15, 3.10
Education	0.41	0.24	8.41	<.001	0.31, 0.50	0.34	0.051	1.68	0.093	–0.05, 0.75	–0.25	–0.02	–0.85	0.391	–0.85, 0.33
Employment Status	0.13	0.08	2.76	<.001	0.04, 0.23	0.56	0.085	2.65	<.001	0.14, 0.98	–0.37	–0.03	–1.19	0.231	–0.98, 0.23
Marital Status	0.03	0.01	0.44	0.657	0.65, 0.21	–0.53	–0.04	–1.42	0.15	–1.26, 0.20	1.25	0.07	2.28	0.023	0.176, 2.32

Abbreviations: Unst:, Unstandardized; St:, Standardized; CI, 95% confidence interval.

Table 7 demonstrates that education increased COVID-19 knowledge (β = 0.411) and attitude (β = 0.34) but was statistically significant only in knowledge (*p*-value < 0.05), While education did not increase practices about COVID-19 (β= –0.26, *p*-value = 0.39) and unsure due to statistically insignificant. Age seemed inverse with knowledge (β= –0.13, *p*-value = –1.64) and positive with attitude (β = 0.20, *p*-value = 0.541) but inconsequential reason was statistically insignificant. In contrast, age significantly improved practices (β = 2.12, *p*-value=<0.05). Gender has a negative and significant relation with knowledge (β= –0.54, *p*-value < 0.05) and attitude (β= –1.14, *p*-value < 0.05), thus practices are negatively correlated with gender (β= –1.85, *p*-value < 0.001). Countries public have different internal knowledge, attitude, and practices of COVID-19. Therefore the impact of the country has a negative but statistically significant result on knowledge (β = –0.20, *p*-value < 0.05) and attitude (β= –1.13, *p*-value < 0.05), but for practices, result are negative and statistically insignificant (β= –0.08, *p*-value = 0.761). Marital status was minor effectual and insignificant with knowledge (β = 0.03, *p*-value = 0.657) and negatively associated with attitude (β= –0.53, *p*-value = 0.15). Whereas, with practices, marital status found positive and significant (β = 1.25, *p*-value < 0.05). Employment positively and significantly absorbed knowledge (β = 0.13, *p*-value < 0.05) and had significant impacts on public attitude (β = 0.56, *p*-value < 0.05), while with respect to practice was negative and insignificant (β= –0.37, *p*-value = 0.231).

## Discussion

This quantitative research is one of the limited studies focusing on emerging countries (China, India, and Pakistan). There has been little research that documents the public knowledge, attitudes, and practices regarding COVID-19, more especially in developing country's perspective. Based on the results shown in Table 6, knowledge score (male = 5.19, and female = 4.63) and criterion of 40%, 40% to 70%, and over 70%. Among the participants, males had higher knowledge than females because males’ were surfing the internet and watched more news related to COVID-19. The results study showed that overall, knowledge, attitude, and practices regarding COVID-19 among adults in three developing countries were found roughly appropriate. However, the attitude and practices were not related to 24.1% of the respondents' knowledge. The large gap was evidenced among people in India and Pakistan related to COVID-19 knowledge, attitude, and practices. These findings of the study in line with the previous study of Honarvar et al.^14^ The authors found the gaps among respondents in the region of Iran. The study results consistent with the study of Abdelhafiz et al.,^21^ in which participants with a lower level of education had less knowledge regarding COVID-19. Also consistent with Alhomoud and Alhomoud,^29^ pilgrims had moderate knowledge related to MERS-Cov. These study findings imply that more efforts should be inputted to deliver a message to illiterate people, which may have financial and media related difficulties to reach accurate information.

The participants' attitudes as a mediator between knowledge and practices have a significant role in better executing and controlling the current epidemic; they support the process of changing individuals' behavior.^26^ This study evidenced that less than half (45.51%) of the participants observed preventive measures regarding COVID-19 in the community of three countries, while in Iran, about 70% observed preventive measures.^14^ Similarly, Usman et al.^34^ observed nearly 60% of preventive measures among the public of South-Western Uganda. Furthermore, over one-third of participants did not agree with government preventives measures, while the public in Saudi Arabia, 98.1%, evidenced the government's precautionary measures. In our study, approximately one-fourth of the people stated that COVID-19 is not a dangerous and deadly disease, whereas in Iran, it's 50%,^14^ and in Thailand, it’s 70%.^35^ Around one-third of the participants stated that media is not playing an adequate role in covey accurate information regarding COVID-19, whereas in developing countries, it's noted 48.8%.^15^ The inaccuracy and exaggeration of pandemic disease information are also confirmed by Mohamad et al.^12^

This study found that three-quarters of participants reported teaching others about the prevention of COVID-19, while in a previous study, it's 60%.^14^ Approximately three-fourth of them started following COVID-19 news, and over 73% practiced 1–1.5 m distance. Furthermore, three-fourth of respondents avoid contact with others. However, half of the respondents reported that they never or rarely wash or disinfect their hands. One-third of the respondents did not wear a mask while going outside, whereas, in New York, it is 80%.^36^ These research findings are weaker than the study by Chen et al.,^37^ who found over 90% of participants wear a mask and avoid going outside. At the same time, these findings of the study are in line with Honarvar et al.^14^

### Practical Implications

This study will help developing countries understand the general public practice and develop strategies to overcome COVID-19. This study will help countries know about their general public's knowledge level, which will help them arrange awareness sessions for the public. This study will also help them identify which group of the general public needs attention and awareness. The study results suggested that government and non-governmental organizations should come at the front to share the information regarding COVID-19 prevention, especially in India and Pakistan. Furthermore, as the disease is dangerous and deadly, people avoid contact with infected persons and crowded places, wash or disinfect their hands, and wear a mask while going outside. Moreover, people are advised not to shake hands and hug others in the current pandemic and keep 1–1.5 m social distance.

### Limitations and Future Recommendations

The strength of the study is that it speeches major health issues that affected the whole world. The limitation of the study includes the convenient sampling and distribution of online surveys in only three developing countries through different social media applications. It allowed only those participants who have internet access, whereas potential respondents may not participate due to internet access. The results of this study provide more valuable insights to governments of three Asian countries when designing future interventions to promote a specific message to improve knowledge, attitude, and enhance practices regarding COVID-19 and the common disease in the future. Furthermore, high-risk participants of communities such as prisoners, elders in hospitals and nursing homes slums, pregnant women, children, patients with chronic disease, disabled persons, barracks staff, socially deprived individuals and citizens of minorities, refugees, and those who are not having equal access to health should not be overlooked in this pandemic as many of them were in the previous pandemic.^38–40^ We recommend conducting a similar research in different developing countries in different regions like Africa to provide a more substantial piece of proof about public knowledge and to detect people with more needs for care and education, etc. Finally, we recommend that the public should follow the instructions of government and health agencies to prevent COVID-19 infections. And, specific to India and Pakistan, even though the government has taken significant measures to prevent the transmission of the disease, substantial effort is required to share accurate information and precautions regarding COVID-19. Besides that, governments in both countries are needed to ensure the implacability of practices among the public.

## Conclusion

Overall, knowledge, attitude, and practices regarding COVID-19 among adults in three developing countries were found roughly appropriate; however, the attitude and practices were not related to 24.1% of the respondents' knowledge. Though most participants know that to control spread and infection from COVID-19 requires avoiding contact with infected persons, less than half of the participants observed with less preventive measures, and nearly half of the respondents showed inadequate behavior towards the disinfection of hands and sanitizing. Furthermore, the appropriate level of knowledge, attitude, and practices regarding COVID-19 evidenced among male participants residing in China, while inadequate found among females, in India and Pakistan, and people aged less and equivalent to 30 years. The attitudes among respondents were found poorer among unmarried people, females, and India and Pakistan residents. While the practices found inappropriate among employed, unemployed and, respondents had a bachelor's degree and females in India. The large gap was evidenced among people in India and Pakistan related to COVID-19 knowledge, attitude, and practices.
